# Multiscale deconstruction of molecular architecture in corn stover

**DOI:** 10.1038/srep03756

**Published:** 2014-01-20

**Authors:** Hideyo Inouye, Yan Zhang, Lin Yang, Nagarajan Venugopalan, Robert F. Fischetti, S. Charlotte Gleber, Stefan Vogt, W. Fowle, Bryan Makowski, Melvin Tucker, Peter Ciesielski, Bryon Donohoe, James Matthews, Michael E. Himmel, Lee Makowski

**Affiliations:** 1 Department of Electrical and Computer Engineering, Northeastern University, Boston, MA 02115; 2 National Synchrotron Light Source, Brookhaven National Laboratory, Upton, NY 11973; 3 GM/CA CAT, XSD, Advanced Photon Source, Argonne National Laboratory, Argonne, IL 60439; 4 X-ray Science Division, Advanced Photon Source, Argonne National Laboratory, Argonne, IL 60439; 5 Department of Biology, Northeastern University, 360 Huntington Avenue, Boston, MA 02115; 6 Department of Physics, Rensselaer Polytechnic Institute, Troy, NY, 12180; 7 Chemical and Biosciences Center, National Renewable Energy Laboratory, Golden, CO, 80401; 8 Department of Chemistry and Chemical Biology, Northeastern University, Boston, MA 02115

## Abstract

Lignocellulosic composite in corn stover is a candidate biofuel feedstock of substantial abundance and sustainability. Its utilization is hampered by resistance of constituent cellulose fibrils to deconstruction. Here we use multi-scale studies of pretreated corn stover to elucidate the molecular mechanism of deconstruction and investigate the basis of recalcitrance. Dilute acid pretreatment has modest impact on fibrillar bundles at 0.1 micron length scales while leading to significant disorientation of individual fibrils. It disintegrates many fibrils into monomeric cellulose chains or small side-by-side aggregates. Residual crystalline fibrils lose amorphous surface material, change twist and where still cross-linked, coil around one another. Yields from enzymatic digestion are largely due to hydrolysis of individual cellulose chains and fragments generated during pretreatments. Fibrils that remain intact after pretreatment display substantial resistance to enzymatic digestion. Optimization of yield will require strategies that maximize generation of fragments and minimize preservation of intact cellulosic fibrils.

Recent attempts to utilize lignocellulose as a feedstock for production of biofuels have focused on identifying enzymes with enhanced hydrolytic capabilities^1^ or pretreatments that provide better access of enzymes to cellulosic fibrils^2^,^3^,^4^,^5^,^6^,^7^. Deconstruction efficiency is limited, however, by the recalcitrance of residual components^8^,^9^, and improving yield requires a focus on the integrity of the cellulose fibrils themselves. Structural studies have provided insight into the nanoscale architecture of lignocellulose^10^,^11^ and its reaction to pretreatments^12^,^13^ but the molecular mechanism of deconstruction and the basis of recalcitrance remain obscure. Here, we study the impact of pretreatment on the micro- to nanoscale architecture of maize cell walls using x-ray scattering over an angular range corresponding to length scales from 2.2 Å to 5000 Å. X-ray fluorescence microscopy (XFM)^14^, transmission (TEM) and scanning electron microscopy (SEM) are used to track specific components and interpret scattering data. We show that cellulose fibril deconstruction during pretreatment is a complex process that disorders and disorients the fibrils. Many fibrils disintegrate into monomeric cellulose chains or small side-by-side aggregates. Yields from enzymatic digestion are largely due to hydrolysis of individual cellulose chains and fragments generated during pretreatments. The differential in digestibility between intact fibrils and fragments generated by pretreatments indicates that improved yield from processing of biomass will require treatments that maximize generation of fragments and minimizes preservation of intact cellulosic fibrils.

Structural studies were carried out on milled, dried maize stover, untreated and after steam explosion pretreatment^2^,^3^ with hot water (H2O); dilute H_2_SO_4_ (DA); dilute acid plus 2 mM Fe_2_(SO_4_)_3_ (DA/Fe); and hot water plus 2 mM Fe_2_(SO_4_)_3_ (H2O/Fe). Yields from enzymatic hydrolysis are given in Table 1 where saccharification yield is given in grams glucose per 100 grams glucan. Materials pretreated with hot water, dilute acid and dilute acid plus iron sulfate exhibit a monotonic progression of saccharification yields.

The morphology of milled maize at the macro scale is not dramatically altered by pretreatment. Grindings retain a clear fiber axis and are often associated with lignin-rich amorphous material extruded from the cellulosic scaffolding^4^. The impact of pretreatments at the 100 nm scale was assessed using ultra small-angle x-ray scattering (USAXS) at beam line X9 at the National Synchrotron Light Source^15^. USAXS from untreated samples exhibited characteristic diamond-shaped diffuse scattering with a sharp spike of intensity along the equator (Figs. 1a, S4). Intensities along these spikes were interpreted as due to a population of cylindrical bundles of lignocellulose exhibiting a Gaussian distribution of diameters (SI and Figs S5; Table S1). In the untreated samples, the average diameter varied from 0.12–0.14 μm with standard deviations of 0.06–0.07 μm. SEM images (Fig. 2a) confirm that these samples are made up of cylindrical bundles with diameters averaging ~ 0.13 μm.

Pretreatment results in virtually no change in the size of the structural elements giving rise to the equatorial streaks (Table S1 and Fig. S5) and SEM results (Fig. 2a) confirm this while revealing distinct changes in the texture of surface structures. Conversely, the diamond-shaped diffuse scatter becomes almost circularly symmetric after pretreatment. The near circular symmetry of this diffuse scatter from pretreated material indicates substantial disorientation of these components on the 100 nm length scale. USAXS patterns from Fe_2_(SO_4_)_3_-pretreated samples exhibit an enhancement of the equatorial streak (Fig. 1a) suggesting that the electron dense iron is efficiently permeating the lignocellulosic bundles. XFM confirmed the uniformity of iron penetration (Figs. 2b, 2c and S3). TEM images of DA- and DA/Fe-pretreated samples exhibit considerable heterogeneity in the degree of deconstruction observed with regions close to the compound middle lamella displaying more nano-fibrilation and delamination than regions deeper in the secondary cell wall (Fig. 2d).

Structural changes at the nanometer scale were assessed by wide-angle x-ray scattering (WAXS) at beam line 23ID-B at the Advanced Photon Source using a 5 μ diameter x-ray beam^16^ (Fig. 1b). These patterns are dominated by a pair of equatorial peaks and a set of sharp meridionals, consistent with scattering from fibrous cellulose crystallites exhibiting a lattice either identical or closely similar to cellulose I_β_. A microscope accommodating a small hole down the optic axis for the x-ray beam provided coaxial visualization of samples and the capability of recording SAXS/WAXS patterns from 1 μm diameter fibers frayed off the main body of biomass particles. A subset of these patterns exhibited one or more sets of sharp reflections in the small angle (SAXS) region (Fig. 1c). Subsidiary reflections were weak, precluding an unambiguous indexing. Assuming they derive from a two-dimensional hexagonal lattice, their positions indicate a lattice with center-to-center spacing of ~ 53 Å. Analogous reflections in scattering from cotton linters indicated a spacing of ~ 71 Å, consistent with the larger size of cellulose fibrils in cotton^17^,^18^. Observations of a peak in the interference function in scattering from primary-wall cellulose in celery collenchymas^11^,^19^ were reported to arise from side-by-side packing of microfibrils, the presumptive origin of the reflections reported here. But peaks of the sharpness observed here are unprecedented and indicate a degree of ordered packing of microfibrils unexpected in dried material. The breadth of these reflections is consistent with ordered arrays approximately 0.07 μ in diameter, large enough to accommodate ~ 130 microfibrils. They were never observed in pretreated samples under any conditions suggesting efficient disruption of the structural elements responsible for maintaining the ordered packing of microfibrils in untreated samples.

Breakdown of the crystalline fibrils was assessed by analysis of equatorial scattering (Fig. 3a). The intensity is dominated by two peaks, one the superposition of (1 1 0) and (1 -1 0) reflections, and the other the (2 0 0). Multiple approaches (see SI) to analyzing these results arrive at the same conclusion: Scattering from DA-pretreated material reflects a substantial decrease in crystallinity compared to control whereas DA/Fe-pretreated material exhibits a crystallinity nearly as high as untreated. Higher crystalline content or crystallinity index (CI) is usually correlated with greater recalcitrance to enzymatic digestion^20^, so why does DA/Fe-pretreated material produce higher saccharification yields? We hypothesize that the crystallinity index of the DA/Fe-pretreated sample is overestimated due to the presence of a large population of individual cellulose chains that exhibits featureless x-ray scattering interpreted as background. DA pretreatment leads to the partial breakdown of cellulose fibrils into side-to-side aggregates of multiple cellulose chains that scatter strongly in the region of the equator used to estimate crystalline content. In DA/Fe-pretreated samples, this material is further broken down into fragments too small to produce substantial scatter in this region. The remaining population of crystalline fibrils is thereby overestimated because of the absence of confounding scatter in the region of the (2 0 0) and (1 1 0)/(1 -1 0) reflections on the equator.

We estimated apparent crystallinity in two independent ways: molecular modeling of the equatorial scattering of the three components; and modeling the equatorial scattering on the basis of a two component system (Figs. 3, S8) in which the scattering from single chains was treated as background. Scattering from single chains was considered in concert with scattering from non-cellulosic materials to make an overall estimate of the amount of material giving rise to ‘background’ scattering (SI). In the two component model, scattering from a two-layer, six-chain aggregate was used as a surrogate for scattering from the highly heterogeneous amorphous fraction; a 36-chain fibril was used to represent scattering from the crystalline fraction. The two-component model fit the data well and led to estimates for crystalline content as ~ 79% of observable cellulose in untreated maize; 39% in DA-pretreated maize; and 69% in DA/Fe-pretreated maize. Alternate methods (SI) resulted in somewhat different absolute numbers but preserved these trends.

The diameter of fibrils estimated from the SAXS region (SI) decreases by about 10% on pretreatment. But the diameter of the crystalline part of the fibrils (estimated from the breadth of the (2 0 0) peak -radial coherence length) exhibits no change in DA samples and a small *increase* in some DA/Fe-pretreated samples (Table S2). These observations indicate that pretreatments remove non-periodic, possibly non-cellulosic, material from the surfaces of fibrils, but do not appreciably alter the radial extent of the crystalline cellulose lattice. Other investigators have reported apparent increases in the radial extent of the fibril lattice after pre-treatments^13^. In light of the corresponding SAXS results, the apparent increase in radial coherence length may be attributable to increased homogeneity of lattice constant rather than an increase in radial extent of the lattice.

WAXS patterns from untreated maize are universally well oriented, exhibiting a single fiber axis (Figs. 1b and S6). Patterns from DA-pretreated material are poorly oriented and those from DA/Fe-pretreated materials often exhibit double orientation. These observations reflect a significant reorientation of cellulosic fibrils in pretreated material that must be accommodated within the ~ 100 nm bundles. Double orientation arising from the spiral twisting of cellulose around plant cells has been widely reported^21^,^22^, but reorientation on the micron length scale does not occur in these samples as evident from the USAXS data. This indicates that the double orientation is due to re-orientation at smaller length scales. One possible origin is coiling of fibrils around one another (Fig. 4a). When cross-linked fibrils alter their twist^23^,^24^,^25^ they will react by coiling around one another^26^,^27^ (Fig. 4b).

These observations provide substantial insight into the mechanisms of pretreatment deconstruction. Data sensitive to nanometer length scales demonstrate that pretreatments break some of the fibrils into heterogeneous fragments (Fig. 4c). DA/Fe-pretreatment removes 75% of the fragments that survive DA-pretreatment while breaking down only 35% of the crystalline fibrillar material. Although apparent crystallinity increases in DA/Fe-pretreatment (compared to DA), the actual crystalline content decreases^28^,^29^ (Fig. 4d). Comparing yield from enzymatic treatments with apparent crystallinity of these samples indicates that less than 20% of the fibrillar material is susceptible to enzymatic digestion and suggests that virtually all saccharification is due to breakdown of sub-fibrillar materials (SI).

Even fibrils that remain intact undergo structural changes. Their amorphous surface layers are stripped while their crystalline portion remains intact. Those that remain cross-linked appear to coil around one another, suggesting that the twist of individual fibrils is changed by pre-treatments. Packing of fibrils around one another becomes more open in the pretreated materials as they spread into space freed by the extrusion of lignin^4^ and disintegration of other fibrils (Fig. 4e).

It has long been recognized that the recalcitrance of fibrils to enzymatic digestion is substantially greater than that of smaller fragments or clusters of cellulose chains^30^. We hypothesize that this difference may be due to the flexibility of the smaller structures. Enzymatic hydrolysis of β(1 → 4) glycosidic bonds between glucose molecules occurs through a multi-step mechanism in which the rate-limiting step is the formation of a carbocation adjacent to the ether oxygen, forming a partial double bond and accompanied by an obligate distortion of the glucose ring^31^,^32^,^33^. Interactions with adjacent chains may resist this distortion even for chains on the fibril surface^34^, increasing the energy barrier of the rate-limiting step and slowing hydrolysis. One- or two-layer fragments formed by pretreatments are more flexible than the intact crystalline fibrils, and might better accommodate ring distortion than the interactions within a rigid, intact fibril. Our observations indicate that virtually all the sub-fibrillar fragments in pretreated materials are digested when subjected to enzymatic hydrolysis whereas less than 20% of fibrillar material undergoes enzymatic digestion in the experiments described here.

Fibrils that avoid disintegration in pretreatments constitute a nearly non-hydrolyzable fraction of cellulose first recognized over 70 years ago^8^,^9^. We conclude that approaches to pretreatment that favor formation of sub-fibrillar fragments over preservation of intact fibrils have the potential to enhance yields substantially beyond those currently achievable.

## Author Contributions

H.I. carried out much of the analysis of USAXS and WAXS data, Y.Z. participated in the analysis of USAXS and SAXS data; L.Y. collected the USAXS data; N.V. and R.F.F. participated in collection of the WAXS data; S.C.G. and S.V. collected the XFM data; W.F. carried out the SEM; B.M. analyzed the consequences of twist and packing on hydrolysis; M.T. prepared the samples; P.C. and B.D. carried out the TEM; J.M. analyzed the twist; M.E.H. and L.M. conceived and designed the study; M.E.H. coordinated sample preparation and electron microscopy; L.M. coordinated all aspects of the project and with the participation of all authors interpreted the data and prepared the manuscript.

## Supplementary Material

Supplementary Information Supplementary Information

## Figures and Tables

**Figure 1:**
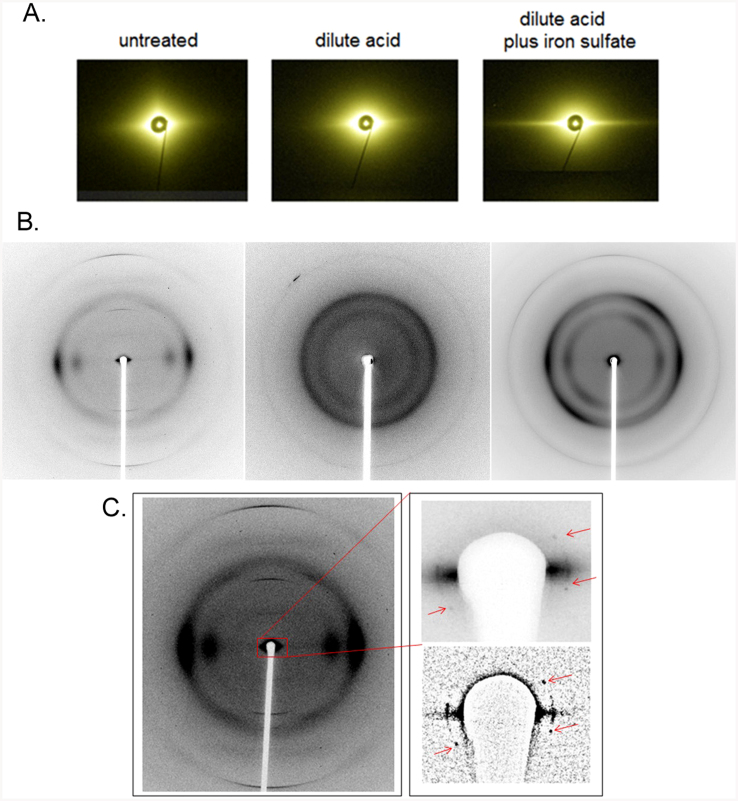
X-ray data. (A) USAXS patterns (left-to-right), untreated; DA-pretreated; DA/Fe-pretreated. (B) WAXS patterns (left-to-right), untreated; DA-pretreated; DA/Fe-pretreated samples. (C) WAXS pattern from a sample exhibiting sharp SAXS reflections observed from very small (<5 μ^3^) scattering volumes of untreated material. Insets show an enlargement of the small angle region (top) and a high-pass filtered version to enhance their visualization (bottom).

**Figure 2:**
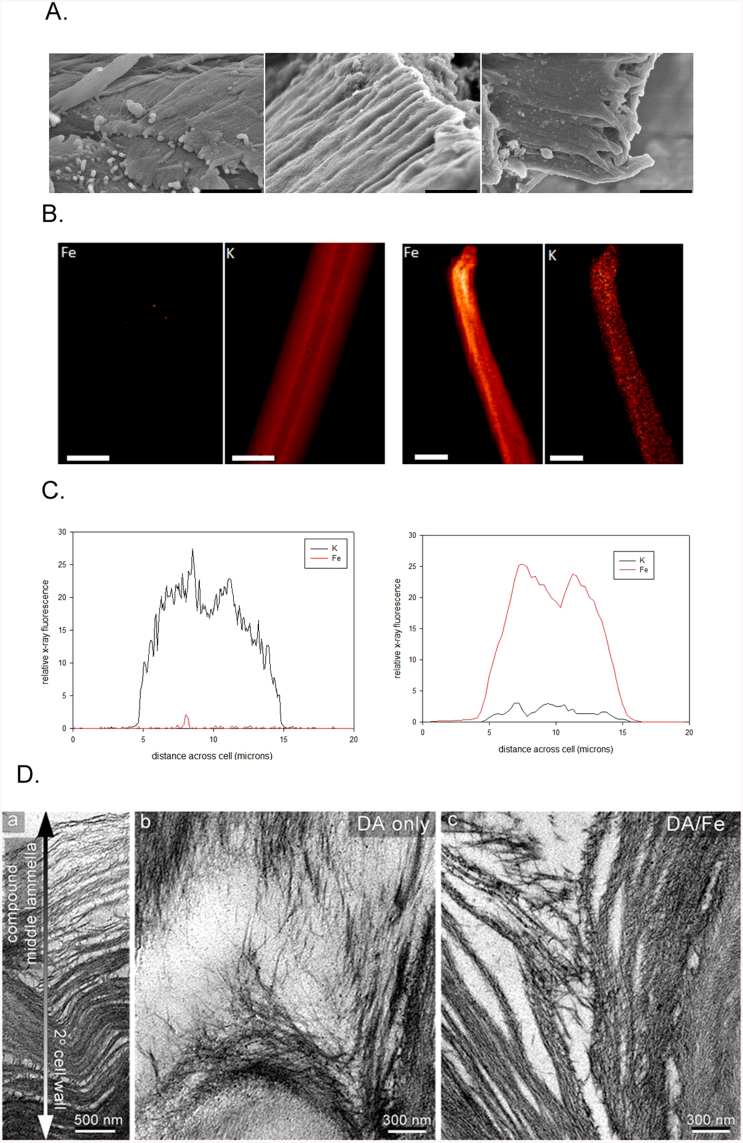
(A) SEM images of (left) untreated; (middle) DA-pretreated maize; (right) DA/Fe-pretreated maize. (Scale Bars = 1.0 μ). (B) X-ray fluorescence images of (left) untreated; (right) DA/Fe-pretreated fiber cells from maize (scale bars = 10 μ). (C) Elemental abundance traces perpendicular to the cells in (B) showing the distribution of iron and potassium in (left) untreated and (right) DA/Fe-pretreated material. Dips near the center of the distributions reflect the uniform distribution of elements through the cell walls of these hollow fiber cells. Images of the distribution of other elements are in Figure S3. (D) TEM images of the middle lamella in untreated (left); DA-(middle) and DA/Fe- (right) pretreated corn stover. Pretreatment results in gradients in degree of deconstruction across the cell wall.

**Figure 3:**
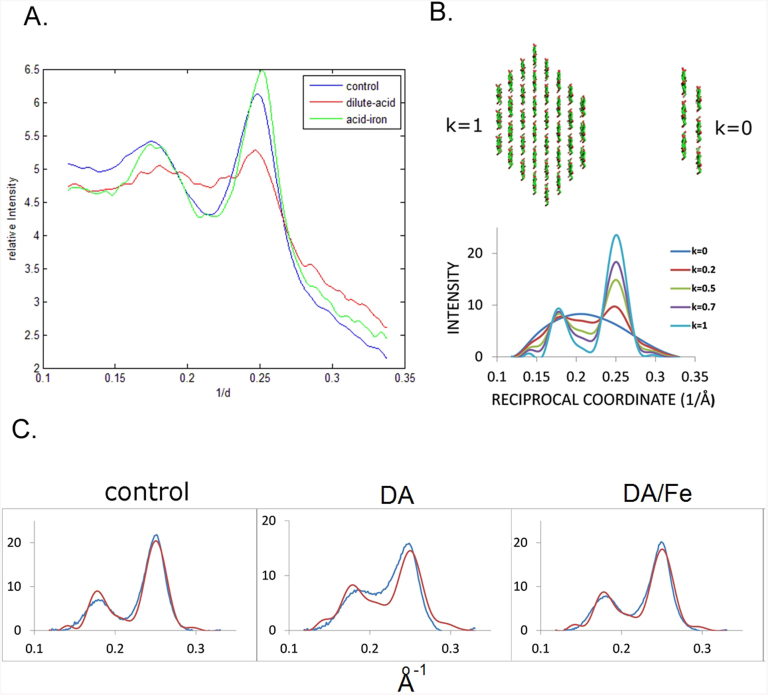
Modeling the equatorial data: (A) Equatorial traces from WAXS patterns of untreated; DA- and DA/Fe-pretreated samples. (B) Equatorial scattering predicted from mixtures of 36-chain fibrils and 2-layer fragments (C) Comparison of background subtracted intensity (blue) with that predicted (red) for scattering from mixtures of 36 chain fibrils and two-layer aggregates. Additional details of the data analysis are included in the SI and Figures S6, S7 and S8.

**Figure 4:**
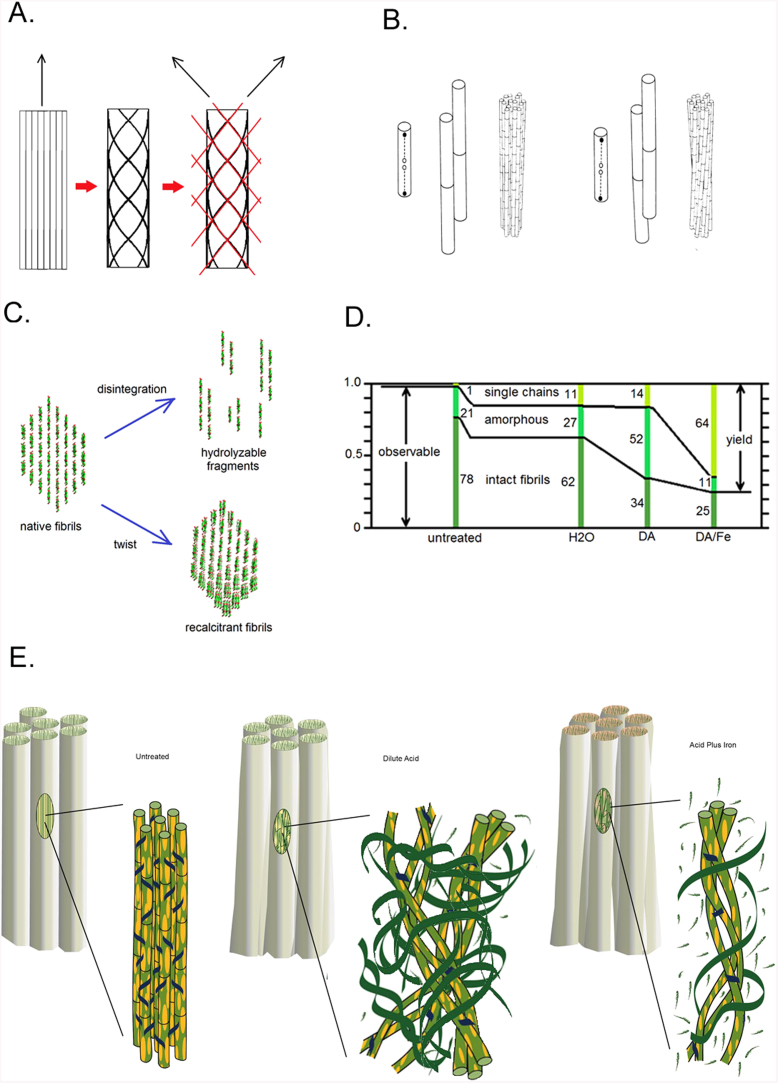
Twisting, coiling and digestion of cellulose fibrils in pretreated materials: (A) Diffraction patterns from bundles of coiled fibrils exhibit double orientation since diffracting geometry emphasizes orientation of fibrils at the front and rear faces of a bundle. Scattering from the front of a right-handed coil will generate a fiber pattern tilted clockwise; scattering from the back will generate a pattern tilted counter-clockwise. Red lines represent the approximate tilt of the meridian in patterns from the front and back of the coiled fibers diagrammed here. (B) Cross-linked fibrils will coil around one another when individual fibrils change their twist (adapted from Weisel *et al*., 1987)^26^. Larger cross-linked aggregates will also coil in response to change in twist of the individual fibrils. (C) Native cellulose fibrils follow one of two pathways during dilute acid pretreatment: They either fragment by slippage of the molecular sheets giving rise individual cellulose molecules or to 1- or 2-layer structures or exhibiting much greater flexibility than intact fibrils; or remain intact, twisting to form rigid, highly recalcitrant coiled fibrils. (D) The relative proportions of three populations of cellulosic materials evolve during pretreatment. The numbers represent percentages of total cellulose (as distinct from apparent crystallinity calculated from the equatorial scattering) calculated assuming fibrils are completely resistant to digestion (see SI). (E) Transformations of nano-scale architecture during pretreatments: Non-cellulosic materials (yellow ellipses) are removed from the fibril surfaces. Cross-linking hemicellulose (blue) is cleaved, releasing the fibrils to twist and, where still cross-linked to coil around one another. Fibrils are progressively deconstructed into fragments (green ribbons) and individual chains (green brush marks) as the harshness of the treatment increases. (Drawing by Karen Moore).

**Table 1:** Yields of Enzymatic Hydrolysis after Pretreatment

Pretreatment	Saccharification yield
H2O	37.6 ± 1.0
DA	66.6 ± 1.2
DA/Fe	74.9 ± 1.5
