# Supplementary material for: Multiscale deconstruction of molecular architecture in corn stover
Source: Sci Rep. 2014 Jan 20;4:3756. doi: 10.1038/srep03756 (PMC3895879; doi:10.1038/srep03756)
Supplement: Supplementary Information [file srep03756-s1.doc]

**Supplementary Materials:**

Multiscale deconstruction of molecular architecture in corn stover

**Authors:** Hideyo Inouye^1^, Yan Zhang^1^,Lin Yang^2^,Nagarajan Venugopalan^3^, Robert F. Fischetti^3^, S. Charlotte Gleber^4^, Stefan Vogt^4^, W. Fowle^5^, Bryan Makowski^6^, Melvin Tucker^7^, Peter Ciesielski^7^, Bryon Donohoe^7^, James Matthews^7^, Michael E. Himmel^7^,

and Lee Makowski,^1,8^*

**Affiliations:**

1. Department of Electrical and Computer Engineering, Northeastern University, Boston, MA 02115.

2. National Synchrotron Light Source, Brookhaven National Laboratory, Upton, NY 11973.

3. GM/CA CAT, XSD, Advanced Photon Source, Argonne National Laboratory, Argonne, IL 60439.

4. X-ray Science Division, Advanced Photon Source, Argonne National Laboratory, Argonne, IL 60439.

5. Department of Biology, Northeastern University, 360 Huntington Avenue, Boston, MA 02115.

6. Department of Physics, Rensselaer Polytechnic Institute, Troy, NY, 12180.

7. Chemical and Biosciences Center, National Renewable Energy Laboratory, Golden, CO, 80401.

8. Department of Chemistry and Chemical Biology, Northeastern University, Boston, MA 02115.

*To whom correspondence should be addressed. E-mail: makowski@ece.neu.edu (L.M.)

**1. Sample preparation:** Samples were prepared as previously described^3^. Briefly, corn stover was prepared from field-senesced corn stems (Round-Up Ready Pioneer hybrid-36N18) hand-cut from a single field (Gustafson Farm, Weld County, CO). Stalks were air dried to a moisture content of 10% prior to dissecting. They were then milled to 20-mesh in a Thomas Wiley (Swedesboro, NJ) cutting mill and subsequently freeze-dried. Samples containing both primary and secondary cell walls were prepared, i.e. dried and milled control corn stover , dried and milled corn stover which was pre-treated (prior to drying and milling) with hot water (150^o^ C for 15 minutes ); with 0.5% H2SO4 (150^o^ C for 15 minutes) and dried and milled corn stover which was pre-treated with either hot water or 0.5 % H2SO4 and 2mM Fe2(SO4)3 at 150^o^ C for 15 minutes. All pretreatments were carried out at the same time in 35 ml heavy walled, glass pressure reactors sealed inside a direct steam injected 2-gal Parr reactor. A separate glass reactor with water and thermocouple was buried in the middle of the set of reactors to monitor temperature. Superheated steam (20°C superheat) was injected first until the temperature of the glass reactors reached within 10°C of desired temperature to rapidly heat the reactors at which time pressure was readjusted lower to the desired saturated steam pressure/temperature of the reaction. Samples were kept at ambient temperature until the scattering experiments. Cotton linters were handled in essentially identical fashion.

The glucose yield from enzymatic hydrolysis of different pretreated corn stover residue is reported as grams glucose yield per 100 grams glucan after 96 h enzymatic digestion.

**2. Optical micrographs: Figure S1** includes representative images of (a) untreated, (b) DA-pretreated and (c) DA/Fe-pretreated maize stover. The gross morphology of most fibrils appeared to change relatively little although some discoloration was apparent, particularly in the DA/Fe-pretreated samples. Pre-treatment led to the appearance of dark colored globular features (identified as lignin-rich material^4^) that appeared to have been extruded in liquid form from the lignocellulose to re-solidify on the surface of the fibrillar framework. Most material appeared to maintain an obvious fiber axis suggesting that pre-treatments led to little disruption of the orientation of structural elements at the 100 μ length scale.

**3. SEM images: Figure S2 (a-c)** includes low resolution images of untreated and DA- and DA/Fe-pretreated samples showing that the oriented, fibrillar gross morphology of sample fragments is not significantly disrupted by the pretreatments. **Figure S2d** is a high resolution SEM showing the appearance of lignin-rich globules at a crack in the surface of a fibril in a DA/Fe-pretreated sample.

**4. XFM Results: Figure S3** includes representative images of (a) untreated and (b) DA/Fe-pretreated maize stover. The upper left-hand images are differential phase contrast images; the remaining images are the distribution of elements throughout the same field of view. Images chosen here are from fiber cells, hollow in the middle. Distribution of elements P, S, Cl, K, Cu and Zn are more or less uniform throughout the lignocellulosic cell wall. Iron is essentially absent in the untreated material, but abundant and evenly distributed throughout the cell wall in the DA/Fe-pretreated samples. Scale bars are 10 microns.

**5. TEM Results: Figure 2(d)** TEM is highly complementary to x-ray scattering in characterization of pretreatment deconstruction. Delamination of cell wall lamellae is clear in many micrographs of pretreated material and tomograms demonstrate the coiling of fibrillar material. TEM images of pretreated material give the impression of a gradual progression from tightly packed material that has been, perhaps, less exposed to pretreatment acids, to more loosely packed, deconstructed material that was more thoroughly penetrated by acid. Whereas the x-ray data is a weighted average of scattering from all structures in the scattering volume, the TEM provides a view of the range of structural re-arrangements that contribute to the x-ray scattering.

**6. USAXS patterns: Figure S4** includes plots of the intensity distribution as a function of angle about the center of the USAXS patterns shown in **Figure 1a**. The pairs of sharp peaks in these curves correspond to the horizontal streak observed in those patterns (which occurs in two positions, 180^o^ apart). In scattering from untreated material, a weak peak half way between the two strong peaks in scattering is observed and arises due to the diamond-shaped background that becomes circularly symmetric in scattering from pretreated materials. A portion of the intensity behind the beam stop is not shown. The Cauchy function (red) was used to fit the observed curve.

**6.1 Size and Shape of ~0.1 μ Bundles of Cellulose:** All evidence is consistent with the equatorial spikes being due to scattering of axially oriented ~ 0.1 μ diameter cylindrical structures greater than 1 μ in length and comprising bundles of cellulose microfibrils. The distribution of diameters of these structures was estimated by modeling the intensity as a function of scattering angle along the streaks. The observed intensity was measured along the radial direction on the CCD image by angular-averaging the chosen image area using FIT2D^35,36^ or tracing the averaged intensities within the rectangular window along the radial direction using the image processing package, Image-J.

**6.2 Solid cylinder model:** The intensity distribution of solid cylinders having different radii may be written as

$I(R,Z)=\{\int_{r=0}^{\infty} [\frac{rJ_{1}(2\pi rR)}{R}]^{2}\frac{1}{\sqrt{2\pi}\sigma_{r}}\exp[-\frac{(r_{0}-r)^{2}}{2\sigma_{r}^{2}}]dr\}[\frac{\sin(\pi LZ)}{\pi Z}]^{2}*f(R,Z)$.

Here R,Z are radial and axial components of cylindrical reciprocal coordinates. The reciprocal axial direction was parallel to the axial direction in the real space. The fiber axis was chosen as the meridional direction. The equatorial streak normal to the axis was interpreted as arising from the solid cylinders aligned along the principal fiber axis.

The streak arises from the first term which is dependent on R, while the Z-direction broadening arises from the length of the solid cylinder in the second term. If the disorientation also contributes to the scattering broadening along the Z-direction the distribution function $f(R,Z)$ of the disorientation may be convoluted. Here a normalized distribution function having the integral width $w=R\beta$ and disorientation angle β was formulated as

$$f(R,Z)=g(R,Z)=\frac{1}{w}\exp(-\pi Z^{2}/w^{2})$$

for a Gaussian distribution, and

$$f(R,Z)=c(R,Z)=\frac{w}{w^{2}+\pi^{2}Z^{2}}$$

for a Cauchy distribution function.

The Fourier transform of a slab having the height L was approximated by a Gaussian function according to

$$\begin{matrix} \frac{\sin^{2}(\pi LZ)}{(\pi Z)^{2}}=L^{2}\sin c^{2}(\pi LZ)\approx L^{2}\exp[-\pi(LZ)^{2}]=Ls(Z) \\ s(Z)=\frac{1}{(1/L)}\exp[-\pi Z^{2}/(1/L)^{2}] \end{matrix}$$

where $s(Z)$is a normalized Gaussian function with the integral width of $1/L$.Then the observed integral width of the intensity distribution along the Z-direction, $\tilde{w}(Z)$, by considering the integral width of the incident beam $B$may be given by

$$\tilde{w}^{2}=(1/L)^{2}+(R\beta)^{2}+B^{2}$$

for a Gaussian distribution, and

$$\tilde{w}=(1/L)+(R\beta)+B$$

for a Cauchy distribution.

A plot of either $\tilde{w}^{2}$or as a function of $R^{2}$ or $R$ should give a straight line with a slope $\beta^{2}$ or$\beta$, and the y-intercept giving either $(1/L)^{2}+B^{2}$ or $(1/L)+B$.

At Z=0 the intensity distribution may be written as

$I(R,0)=\{\int_{r=0}^{\infty} [\frac{rJ_{1}(2\pi rR)}{R}]^{2}\frac{1}{\sqrt{2\pi}\sigma_{r}}\exp[-\frac{(r_{0}-r)^{2}}{2\sigma_{r}^{2}}]dr\}$.

The observed intensity distribution on the equator may be compared with the calculated intensity according to the above equation. The minimum residual may be searched by systematically changing the average radius of the solid cylinder and its standard deviation.

The size, shape and orientation implied by the equatorial streak in the USAXS scattering patterns are consistent with a highly oriented population of ligno-cellulosic bundles that have been visualized by AFM, TEM and other microscopies and are clearly seen in SEM (**Figure 2a**). Use of USAXS for their characterization facilitates a quantitative, statistically significant analysis of the population of structures *in situ* and provides a broader survey of average characteristics than possible with microscopy.

The resulting estimates for average radius and standard deviation of radius for untreated, DA- and DA/Fe- pretreated materials are tabulated in **Table S1**.

**6.3 Length and Orientation of ~0.1 μ Bundles:** The length and degree of orientation of the structures giving rise to the equatorial streaks in the USAXS patterns (**Figure 1 and S4**) were estimated by modeling the intensity distribution across the breadth of the streaks (**Figures S5** and **Table S1**). The plot of integral width as a function of scattering angle resulted in a straight line with intercept displaced from the origin indicating that the observed integral width was a result of contributions due to both the finite length of the structures and the degree to which they are disoriented in the material^37^. In plots of integral width as a function of reciprocal space coordinates for three different samples of untreated, DA-pretreated and DA/Fe-pretreated samples the y-intercepts provided a measure of the length of the structures and its slope a reflection of the disorientation. The intercepts of all plots of equatorial streaks indicated that the structures giving rise to this scatter are greater than 1 µ in length. The variation of slope observed over different portions of the samples suggest local variation in cellular anatomy gives rise to substantial variation large enough to obscure any effect of pretreatment on the orientation of these bundles (**Table S1**).

**Figure S5** provides an example of the variation of R-factor as a function of the change in average radii and standard deviation of radii in modeling. The contour plot demonstrates that the data is sufficient to constrain estimates of average radii to better than +/- 50 Å and standard deviation to less than +/- 25 Å.

**7. SAXS/WAXS patterns:** SAXS/WAXS patterns were collected from multiple fibrils collected from different sample fractions generated months apart. Over a dozen diffraction patterns were collected for untreated; DA-pretreated; and DA/Fe-pretreated samples. Smaller numbers of patterns were collected from H2O- and H2O/Fe-pretreated materials. **Figure S6** includes representative diffraction patterns. Equatorial intensities were estimated by averaging intensities onto a polar coordinate system (δq,δθ) where dθ was chosen to be approximately the half-width of disorientation in the (2 0 0) reflection. Background beneath the equatorial reflections was estimated by fitting a polynomial to the intensities on either side of the principal equatorial reflections as seen in **Figure S7** and geometric correction for the disorientation was applied to the background-subtracted intensities.

**7.1 Modeling the SAXS portion of the patterns:** The small angle portion of the pattern was modeled in a manner analogous to that of the USAXS data. In particular, it was modeled as due to a population of cylinders (in this case representing fibrils) characterized by an average radius and standard deviation of radii. The electron density distribution in three-dimensional real and reciprocal coordinates, $\bar{r}$ and $\bar{R}$ , may be given by

where $\rho(\bar{r})$ is electron density at $\vec{r}$, $\tilde{\rho}^{2}(\bar{r}$ is auto-correlation function, and the Fourier transform of the electron density of atom $\rho_{j}(\bar{r})$ gives the atomic factor $f_{j}(\bar{R})$ and the transform of the autocorrelation function gives the intensity function $I(\bar{R})$.

Cylindrical averaging of the exponential term around the cylindrical axis parallel to the $z$in real and $Z$ in reciprocal coordinates gives

where $r$and $R$are radial components of the real and reciprocal cylindrical coordinates, and $\varphi$, $\Phi$are the angular components.

Then the cylindrically averaged intensity may be given by

$<I(R,Z)>=[\sum_{j} \sum_{k} f_{j}(R,Z)f_{k}(R,Z)J_{0}(2\pi r_{jk}R)]\exp i2\pi z_{jk}Z$.

In the current study the equatorial intensity at $Z=0$ was calculated. Using this model, intensities in the SAXS region of the WAXS patterns were used to estimate the diameters of the cellulosic fibrils using a modeling procedure exactly analogous to that used for the USAXS data. The fibrils were modeled as population of cylinders with an average radius and a standard deviation. When this was carried out, the results were:

sample average std. dev

diameter (Å) of diameter (Å)

untreated 31 2

DA 26 6

DA/Fe 27 5

In all cases the apparent diameter of fibrils decreased after pre-treatment and the standard deviation increased. When a simple cylindrical model was used to model this data both the absolute values and observed trends remained very nearly the same.

**7.2 Coherence length:** A second method for estimating the diameter of the fibrils is to utilize the width of the lattice reflections on the equator. These reflections can provide an estimate of the 'coherence length' of the lattice in the radial direction (using the (2 0 0) reflection) and axial direction (using the (0 0 4)). The coherence length differs from the diameter estimated with SAXS data. SAXS data provides an estimate of the total diameter of the fibrils. The coherence length, however, is a measure of the size of the crystalline portion of the structure and is insensitive to, for instance, disordered material on the fibril surface.

A general expression for width of a reflection involves the lattice and shape function, lattice disorder and diffuse scattering terms. According to paracrystalline theory^37-41^ the total intensity, *I,*  may be written as

where the symbols < > and * are the averaging and convolution operations. The first term corresponds to diffuse scattering and the second term corresponds to the Bragg reflections. $Z(\bar{R})$ is the interference function, arises from the Fourier transform of the step function expression for the extent of the lattice, $F(\bar{R})$ is the Fourier transform of the unit cell, $N$is the number of lattice points, *v* is the unit cell volume and $b(\bar{R})$ is the shape function fit to the direct beam. In fiber or powder diffraction, the intensity $I(\bar{R})$ is further cylindrically or spherically averaged. The one dimensional form may be given by

$$I(R)=b(R)*\{N[<F^{2}(R)>-<F(R)>^{2}]+1/d<F(R)>^{2}[Z(R)*\left|\sum(R)\right|^{2}]\}$$

where $d$ is the one dimensional lattice period. The interference function $Z(R)$ is written as

$$Z(R)=[1-H^{2}(R)]/[1+H^{2}(R)-2\left|H(R)\right|\cos(2\pi dR)]$$

where $H(R)=\exp(-2\pi^{2}R^{2}\Delta^{2})$ is the Fourier transform of the Gaussian probability function $h(r)$ used as a representation of the distribution function for the nearest neighbors with standard deviation $\Delta$. The $\left|\sum(R)\right|^{2}$is approximated as $\left|\sum(R)\right|^{2}=(Nd)^{2}\sin^{2}(\pi RNd)/(\pi RNd)^{2}$. Since the diffuse scattering intensity and the structure amplitude change gradually as a function of the reciprocal coordinate, the integral width of the Bragg reflection $w$ may be approximated as

$w^{2}=b^{2}+(1/Nd)^{2}+(\pi^{4}h^{4}/d^{2})(\Delta/d)^{4}$,

where $b$ is the integral width of the direct beam , Nd is the coherence length, $h$ refers to the $h$*th* order reflection and the second and third terms are integral widths of $\left|\sum(R)\right|^{2}$and *Z(R),* respectively. We assume the disorder in the lattice, Δ, to be small enough to be neglected in these calculations allowing the last term to be dropped from the expression. The integral width of the Bragg reflection was measured from the equatorial traces obtained using the data processing package FIT2D^35,36^ and the beam width estimated from traces generated using the program ImageJ. Positions and coherence lengths of the (2 0 0) and (0 0 4) reflections are listed in **Table S2** for untreated and four types of pretreated samples. The results from the (2 0 0) are a measure of the radial extent of the fibrils; the (4 0 0) provides a measure of the spatial coherence along the length of the fibrils. Pretreatments do not lead to a decrease in the radial coherence length and, in the case of DA/Fe, leads to a small increase. These numbers are consistent with the results of model fitting of the 36-chain model described below.

**7.3 Crystallinity Index:** The proportion of cellulose in crystalline (fibrillar) form (sometimes referred to as 'crystallinity index') has been estimated in a number of ways^42^ from x-ray and NMR data. In general, different methods arrive at similar relative numbers although the absolute numbers vary. Here we use two methods to estimate the relative amounts of crystalline cellulose in the samples. In the first, we estimate the total intensity due to crystalline and amorphous fractions; in the second, we use molecular modeling of the x-ray scattering along the equator. As we show in the final sections (below), crystallinity calculated in this way results in an estimate of the *apparent* proportion of crystalline cellulose in a sample. Cellulose present as single chains or as single-layers is relatively featureless in the region of the scattering pattern used to make these estimates and is generally treated as background. This may result in an overestimate of the proportion of cellulose in fibrillar (crystalline) form in samples. In the third approach, we take this component into account to arrive at estimates for the relative abundance of each component.

**7.3.1 Estimate #1:**

Intensities on the equator in the range 0.12 -0.33 Å^-1^ were used for these calculations. Scattering from non-cellulosic material was approximated by a polynomial background (see **Figure S7**) that was subtracted from observed, and the total intensity remaining $I_{t}$, was assumed to be composed of the intensities from the crystalline fraction, $I_{c}$, and amorphous fraction, $I_{a}$, that is $I_{t}=I_{c}+I_{a}$.

The total intensity is then written as

$I_{t}(R)=kI_{c}(R)+(1-k)I_{a}(R)$,

where k is the proportion of cellulose in the crystalline fraction. The correspondence between calculated and observed was assessed on the basis of the $R$-factor where $R=\frac{\sum\left|I_{t}-I_{obs}\right|}{\sum\left|I_{obs}\right|}$.

The basis of the first estimate in the diffracting power *P* from the sample, defined by

$P_{f}=\int I_{f}(\overrightarrow{R})d\overrightarrow{R}$,

for any fraction, *f*, where $\vec{R}$ is the reciprocal coordinate. Parseval's equation relates this to the electron density distribution:

$P_{f}=\int\rho_{f}(\overrightarrow{r})^{2}d\overrightarrow{r}$.

Using the average electron density $<\rho_{t}>$ and the total volume of the scattering object $V_{t}$ the diffracting power can be described as $P=V_{t}<\rho_{t}>^{2}$. Since $P_{t}$is the sum of the crystalline domain (*c*) and the amorphous domain (*a*), i.e. $P_{t}=P_{c}+P_{a}$*,* the volume and the average electron density of the crystal and amorphous domain are related by

$V_{t}<\rho_{t}>^{2}=V_{c}<\rho_{c}>^{2}+V_{a}<\rho_{a}>^{2}$.

Scattering from the amorphous and crystalline fractions were estimated using polynomial fits for background with and without constraining the fit at the mid-point between the two principal equatorial reflections (**Figure S10**). The $P_{c}$was measured from the integral area under the observed intensity curve after subtraction of the amorphous scattering. Assuming the same average electron densities for both crystal and amorphous domains, the volume fraction of crystal domain, $\tilde{V}_{c}$ is calculated according to $\tilde{V}_{c}=P_{c}/P_{t}$. Crystallinity calculated in this way is given in Table S2 for all samples and compares favorably with estimates made on the basis of modeling described below.

**7.3.2 Estimate #2:**

For the second estimate, the fibrillar (crystalline) and sub-fibrillar (amorphous) components were modeled on the basis of the crystal structure of cellulose I_β_ ^43^ and models of elementary fibrils of different sizes and shapes that have been proposed as major components of plant cell walls. The observed peak positions suggest a lattice with dimensions slightly adjusted from the prototypical cellulose Iβ. The observed spacing of the first peak was smaller than the calculated one, and that of the second peak was larger. The observations were consistent with a=7.9 Å, b=7.6 Å, c=10.38 Å and γ=90° (compared to a=7.78 Å, b=8.20 Å, c=10.38 Å and γ=96.55° in the classic I_β_ form).

The crystalline fraction was modeled as a hexagonal fibril containing 36 cellulose chains^44^, and a diamond or rectangular fibril containing 24 chains^10,11^ using the atomic coordinates reported for the I_β_ cellulose^43^ and built using crystalline lattices having either γ=90° or 96.55°. While both hexagonal 36-chain and diamond 24-chain models gave two intensity maxima similar to the ones observed, the rectangular 24 chain model and 18-chain models gave reflections inconsistent with the observations. Consequently, a hexagonal 36-chain model was used for estimating crystalline content.

None of the models for the crystalline fraction predicted the relatively high intensity observed between the (2 0 0) and (1 -1 0)/(1 1 0) reflections at a spacing of about 1/5 Å (**Figure S7**) and attributable to amorphous, sub-fibrillar aggregates of cellulose. Many alternate models for this amorphous fraction were tested. Two-layer models containing 4-7 chains led to intensities predicted scattering most consistent with that observed. Single cellulose chains and fragments constituting a single layer do not scatter in the region. Two-layer fragments are predicted to scatter in quite similar fashions and we demonstrated that, although this population is certain to be structurally diverse, essentially all of the observed scatter could be explained if we approximated the scattering from fragments (amorphous cellulose) as due to a two-layer, six-chain fragment of cellulose (**Figures S8**). We went on to fit all observations to a two-component model consisting of a mixture of 36-chain fibrils and 6-chain fragments.

The intensity based on the two component model was calculated as a function of k- coefficient (where k is the proportion of crystalline cellulose and was compared with the observed intensity. By searching for the minimum R-factor (**Figure S8**) the best k-coefficient was obtained for each sample. Three different crystalline models, i.e. hexagonal 36 chain, diamond 24 chain and rectangle 24 chain models, were tested against the observed intensity after background subtraction (**Tables S2 and S3**). The R-factors demonstrated that the hexagonal 36 chain model gave the best agreement to the data. The k-coefficients for different pretreated samples were determined as 0.84 (control), 0.63 (H2O), 0.63 (H2O/Fe), 0.48 (DA) and 0.71 (DA/Fe), (**Table S3**). These compared well with the first estimate, given in **Table S2**.

**8. Estimate of the proportion of fibrillar material susceptible to enzymatic digestion:** We combined the estimates for crystalline content made by analysis of the equatorial scattering (using method #2 above) with yield measurements to derive a self-consistent set of relative abundances for single chain; amorphous; and crystalline cellulose. The apparently high crystallinity observed in the DA/Fe-pretreated material is due to the presence of a substantial fraction of cellulose as single molecular chains or single layers of chains that contribute scattering difficult to distinguish from background to the portion of the diffraction pattern used to estimate crystallinity. We combined the estimates of crystallinity (from method #2 above) with the observed yields from enzymatic saccharification to make estimates of the proportion of single-chain, amorphous and fibrillar cellulose in each sample as follows: Designate the proportion of single-chain and amorphous cellulose that is digested by R_1_ and the proportion of the fibrillar cellulose enzymatically digested by R_2_. We then have,

Yield = R_1_(S + A) + R_2_ · F

where S, A and F are the proportions of single-chain, amorphous and fibrillar (crystalline) cellulose and

S + A + F = 1.0

Apparent crystallinity, %xtal, is given by

%xtal = F/(A+F)

since the scattering from single chains (S) does not contribute to the portion of the diffraction pattern used to calculate crystallinity. These are three equations in three unknowns (S, A and F), solvable given the observations of Yield and %xtal and estimates of R_1_ and R_2_. Unfortuntely, R_1_ and R_2_ are also unknown. Therefore, we calculated S, A and F for all possible values of R_1_ and R_2_ (0 to 1.0) for all samples. Acceptable values for R_1_ and R_2_ are limited to those that give physically meaningful values for S, A and F (each of which must be in the range 0.0 to 1.0). Values for the untreated material limit R_1_ and R_2_ more than those for other samples. If we assume that there is a very small amount of single-chain cellulose (~ 1%) in the untreated material (as generally believed), we find R_2_ = 0.28 - 0.26·R_1_. If all the amorphous material is digested (R_1_ = 1.0), we conclude that R_2_ = 0.02. That is, if we assume that all the amorphous material in the untreated sample is susceptible to hydrolysis, then we must conclude that the fibrillar material is virtually indigestible. Similarly, if 90% of the amorphous material is digested (R_1_ = 0.9), we conclude that 5% of the fibrillar material is digested (R_2_ = 0.05); if 80% of the amorphous material is digested (R_1_ = 0.8), R_2_ = 0.07. Similarly, for R_1_ = 0.6, R_2_ = 0.12. These numbers strongly suggest that less than 10% of the fibrillar material is susceptible to enzymatic digestion in these samples and establish 20% digestability as an extreme upper bound for the percentage of fibrils susceptible to enzymatic digestion. In other words, our data indicate that most of the amorphous cellulose undergoes enzymatic digestion and most of the fibrillar (crystalline) cellulose does not. If R_1_ and R_2_ are known, it is possible to estimate the amount of single-chain cellulose in each sample. For the case of R_1_=1.0 and R_2_=0.0 we have tabulated the predicted amounts of single-chain, amorphous, and fibrillar (crystalline) cellulose in all the samples analyzed here. These numbers are used for the diagram in **Figure 4e**:

% of observable % of total

cellulose cellulose

population UNT H2O DA DA/Fe UNT H2O DA DA/Fe

single chains -- -- -- -- 1.5 10.8 14.4 63.7

amorphous 21 30 61 31 20.7 26.7 52.2 11.2

fibrils 79 70 39 69 77.8 62.4 33.4 25.1

This calculation indicates that the amorphous fraction comprises over half the observable cellulose in DA-pretreated material but in DA/Fe-pretreated material it breaks down further into chains that do not contribute to scattering in this region, thereby resulting in the anomalously high crystallinity estimate.

**9. Alternative models for the equatorial scattering:** The intensity distribution at the (1 -1 0)/(1 1 0) and the (2 0 0) reflections has been calculated in a number of ways^45,46^. An asymmetric disorder model for the cellulose crystal has proven capable of accounting for the observed equatorial scattering from untreated spruce^9^ and celery^10^. Although this model could also account for our data after background subtraction from untreated samples, it cannot account for the much larger 'diffuse' scattering observed in the pretreated materials. Applying different models to the different samples, or even hybrid models including both asymmetric disorder and amorphous components of cellulose are, of course, possible. But the capability of accounting for diffraction from all samples by the two-component model^47,48^ is quite compelling. Disorder (within individual fibrils) alone seems unlikely to account for all of our observations. It would require us to propose an increase in disorder in response to DA treatment followed by a decrease in disorder after DA/Fe treatment. This seems unlikely.

**Supplementary References**

35. Hammersley, A.P. (1997). FIT2D: an introduction and overview. ESRF Internal Report, ESRF97HA02T.

36. Hammersley, A.P. (1998). FIT2D Reference Manual. ESRF Internal Report, ESRF98HA01T.

37. Guinie,r A. (1963) *X-ray Diffraction.* W. H. Freeman and Co., San Francisco.

38. Inouye, H., Fraser, P.E., Kirschner, D.A. (1993) Structure of beta-crystallite assemblies formed by Alzheimer beta-amyloid protein analogues: analysis by x-ray diffraction. *Biophys. J*. 64: 502-519.

39. Hosemann, R., Bagchi, S.N. (1962) *Direct Analysis of Diffraction by Matter.* North Holland, Amsterdam.

40. Inouye, H. Karthigasan, J., Kirschner, D.A. (1989) Membrane structure in isolated and intact myelins Biophys. J. 56: 129-137.

41. Inouye, H. (1994) X-ray scattering from a discrete helix with cumulative angular and translational disorders *Acta Cryst.* A50: 644-646.

42. Park, S., Baker, J.O., Himmel M.E. Parilla P.A. and Johnson D.K. Cellulose crystallinity index: measurement techniques and their impact on interpreting cellulase performance *Biotech for Biofuels*, 3: 10-19 (2010).

43. Ding, S.-Y., & Himmel, M.E. The maize primary cell wall microfibril: A new model derived from direct visualization. J. Agricultural and Food Chem. 54, 597-606 (2006).

44. Nishiyama, Y., Langan, P. & Chanzy, H. Crystal structure and hydrogen-bonding system in cellulose Iβ from synchrotron X-ray and neutron fiber diffraction. J Am Chem Soc 124:9074–9082 (2002).

45. Nishiyama, Y., Johnson, G. P., and French, A. D. (2012) Diffraction from nonperiodic models of cellulose crystals. Cellulose 19, 3190336.

46. French, A.D., and Cintrón, M.S. (2013) Cellulose polymorphy, crystallite size, and the Segal crystallinity index. Cellulose 20, 583-588.

47. Kawakubo, T., Karita, S., Araki, Y., Watanabe, S., Oyadomari, M., Takeda, R., Tanaka, F., Abe, K., Watanabe, T., Honda, Y., and Watanabe, T. (2010) Analysis of exposed cellulose surfaces in pretreated wood biomass using carbohydrate-binding module (CBM)- cyan fluorescent protein (CFP). Biotechnology and Bioengineering. 105, 499-508.

48. Fox, J.M., Jess, P., Jambusaria, R.B., Moo, G.M., Liphardt, J., Clark, D.S., and Blanch, H.W. (2013) A single-molecule analaysis reveals morphological targets for cellulase synergy. Nature Chemical Biology. 9, 356-361.

**Table S1. Structural parameters derived from USAXS data**

|  | **Average Radius (nm)** | **SD of Radius (nm)** | **R-factor** | **m** | **b [10^-5^]** |
| --- | --- | --- | --- | --- | --- |
| **untreated** | 60 (11) | 38 (5) | 0.050 | 0.74 (.05) | 8 (8) |
|  | 70(8) | 33(6) | 0.052 | 0.79 (.03) | 11 (5) |
|  | 70(9) | 35(5) | 0.052 | 0.52 (.02) | 15 (3) |
| **DA** | 70(17) | 30(8) | 0.061 | 0.94 (.02) | -9 (3) |
|  | 70(15) | 29(5) | 0.074 | 0.89 (.06) | 1 (10) |
|  | 80(10) | 31(5) | 0.076 | 0.97 (.01) | -3 (2) |
| **DA/Fe** | 70(12) | 31(9) | 0.060 | 0.91 (.05) | -7 (10) |
|  | 70(8) | 37(6) | 0.057 | 0.48 (.04) | -2 (10) |
|  | 70(8) | 31(5) | 0.066 | 0.70 (.03) | 3 (7) |

Average radius of cellulosic fibril bundles as derived from USAXS data. Data indicated that the samples were constituted of an ensemble of cylinders which we characterized by an average radius and a standard deviation that provides a measure of the variability of radius. Uncertainties in all parameters are indicated by numbers in parentheses.

Three independent scattering measurements were taken for each sample, indicated by subscripts. The R-factor was defined according to $R=\frac{\sum\left|I_{obs}-I_{cal}\right|}{\sum I_{obs}}$where $I_{obs}$and $I_{cal}$were observed and calculated intensity respectively. The uncertainties in radius and SD of radius (in parentheses) were estimated from the standard deviation of the R-factor as approximated by a Gaussian function. Here the integral width $\delta_{w}$ was related to the standard deviation $\sigma$ by $\delta_{w}=\sigma\sqrt{2\pi}$. The integral width as a function of reciprocal coordinates was fit by a straight curve of$f(x)=m\bullet x+b$ where m is a measure of the disorientation of the cylindrical structures and b a measure of their length. Negative values of b are not physically meaningful, reflect errors in estimation of the intercept and are indications of structures too long for accurate length estimation (> 1.0 μm).

Table S2. Bragg spacings and coherent lengths for pretreated maize samples

| **Sample** | **(2 0 0) (Å)** | **CL(Å)** | **(0 0 4) (Å)** | **CL (Å)** | **Crystal content** |
| --- | --- | --- | --- | --- | --- |
| **untreated** | 4.016 | 32.61 | 2.582 | 244.6 | 0.806 |
|  | 4.010 | 32.26 | 2.576 | 253.8 | 0.772 |
|  | 4.005 | 32.95 | 2.583 | 255.0 | 0.784 |
| **Average** | **4.010(4)** | **32.6(2)** | **2.581(4)** | **250(30)** | **0.79(1)** |
| **H2O** | 4.013 | 31.30 | 2.599 | 245.6 | 0.624 |
|  | 4.023 | 31.39 | 2.594 | 194.6 | 0.638 |
| **Average** | **4.017(5)** | **31.3(1)** | **2.596(2)** | **220(25)** | **0.63(1)** |
| **H2O/Fe** | 4.022 | 33.00 | 2.603 | 207.9 | 0.6336 |
|  | 4.026 | 32.83 | 2.598 | 196.4 | 0.6052 |
|  | 4.047 | 30.60 | 2.599 | 216.4 | 0.6673 |
| **Average** | **4.031(10)** | **32.1(10)** | **2.600(2)** | **207(7)** | **0.64(2)** |
| **DA** | 4.042 | 34.84 | 2.566 | 296.2 | 0.321 |
|  | 4.058 | 30.00 | 2.570 | 196.2 | 0.435 |
|  | 4.037 | 32.89 | 2.581 | 175.6 | 0.416 |
| **Average** | **4.045(8)** | **32.6(17)** | **2.572(5)** | **223(49)** | **0.39(5)** |
| **DA/Fe** | 4.009 | 34.86 | 2.570 | 264.2 | 0.752 |
|  | 4.016 | 34.24 | 2.600 | 239.0 | 0.652 |
|  | 4.011 | 34.64 | 2.598 | 215.7 | 0.678 |
| **Average** | **4.011(3)** | **34.6(2)** | **2.584(14)** | **246(18)** | **0.69(4)** |

Lattice spacings for the (2 0 0) and (0 0 4) reflections , the coherence lengths (CL) calculated from the integral widths of those reflections and the crystallinity calculated as described in the text. The coherent length (Nd) was measured according to $(1/Nd)^{2}=w_{obs}^{2}-b^{2}$ where $N$ is the number of lattice points, $d$ is the lattice spacing, *b* is the integral width of the direct beam, and $w_{obs}$is the observed integral width of the reflection. The mean error is indicated in parenthesis. The crystal content is defined by the ratio between the integral intensity of the crystal domain to that of the total domain in the reciprocal range from 0.12 Å^-1^ – 0.33 Å^-1^.

Table S3. Crystal content coefficient and R-factor with different two basis sets

| **Sample** | **36- & 6-chain** | | **24-diamond & 6-chain** | | **24-rectangle & 6 chain** | |
| --- | --- | --- | --- | --- | --- | --- |
|  | k | R-factor | k | R-factor | k | R-factor |
| **Control** | 0.87 | 0.172 | 0.96 | 0.222 | 0.89 | 0.364 |
|  | 0.82 | 0.169 | 0.89 | 0.237 | 0.85 | 0.372 |
|  | 0.82 | 0.158 | 0.88 | 0.229 | 0.84 | 0.346 |
| **Average** | **0.84(2)** | **0.166(5)** | **0.91(3)** | **0.230(5)** | **0.86(2)** | **0.361(9)** |
| **H2O** | 0.62 | 0.176 | 0.67 | 0.236 | 0.65 | 0.285 |
|  | 0.65 | 0.182 | 0.69 | 0.228 | 0.67 | 0.323 |
| **Average** | **0.63(1)** | **0.179(2)** | **0.68(1)** | **0.232(4)** | **0.66(1)** | **0.304(19)** |
| **H2O/Fe** | 0.64 | 0.171 | 0.69 | 0.224 | 0.63 | 0.333 |
|  | 0.61 | 0.168 | 0.67 | 0.215 | 0.57 | 0.328 |
|  | 0.65 | 0.255 | 0.68 | 0.302 | 0.61 | 0.402 |
| **Average** | **0.63(2)** | **0.198(38)** | **0.68(1)** | **0.246(36)** | **0.60(2)** | **0.354(31)** |
| **DA** | 0.42 | 0.288 | 0.49 | 0.320 | 0.21 | 0.378 |
|  | 0.53 | 0.301 | 0.62 | 0.338 | 0.43 | 0.417 |
|  | 0.48 | 0.274 | 0.56 | 0.315 | 0.28 | 0.384 |
| **Average** | **0.48(4)** | **0.288(9)** | **0.56(4)** | **0.324(9)** | **0.31(8)** | **0.393(16)** |
| **DA/Fe** | 0.73 | 0.161 | 0.78 | 0.248 | 0.77 | 0.312 |
|  | 0.70 | 0.164 | 0.71 | 0.226 | 0.69 | 0.321 |
|  | 0.71 | 0.159 | 0.74 | 0.228 | 0.72 | 0.331 |
| **Average** | **0.71(1)** | **0.162(2)** | **0.74(2)** | **0.234(9)** | **0.73(3)** | **0.322(6)** |

The amorphous component was modeled as a two-layered 6 chain fragment. Three different crystal domains were considered, i.e. the elementary fibril containing 36-cellulose chains^18^, diamond 24 chains^19^ and rectangle 24 chains^19^. The scaling parameter refers to the crystal content coefficient k, and the agreement between the observed and calculated intensities were measured by R-factor (see method).

**Figure S1**

**Figure S1** Optical micrographs of untreated (left), DA-pretreated (middle) and DA/Fe-pretreated (right) maize taken between crossed polars using a 10x objective. Scale bars are 100 microns.


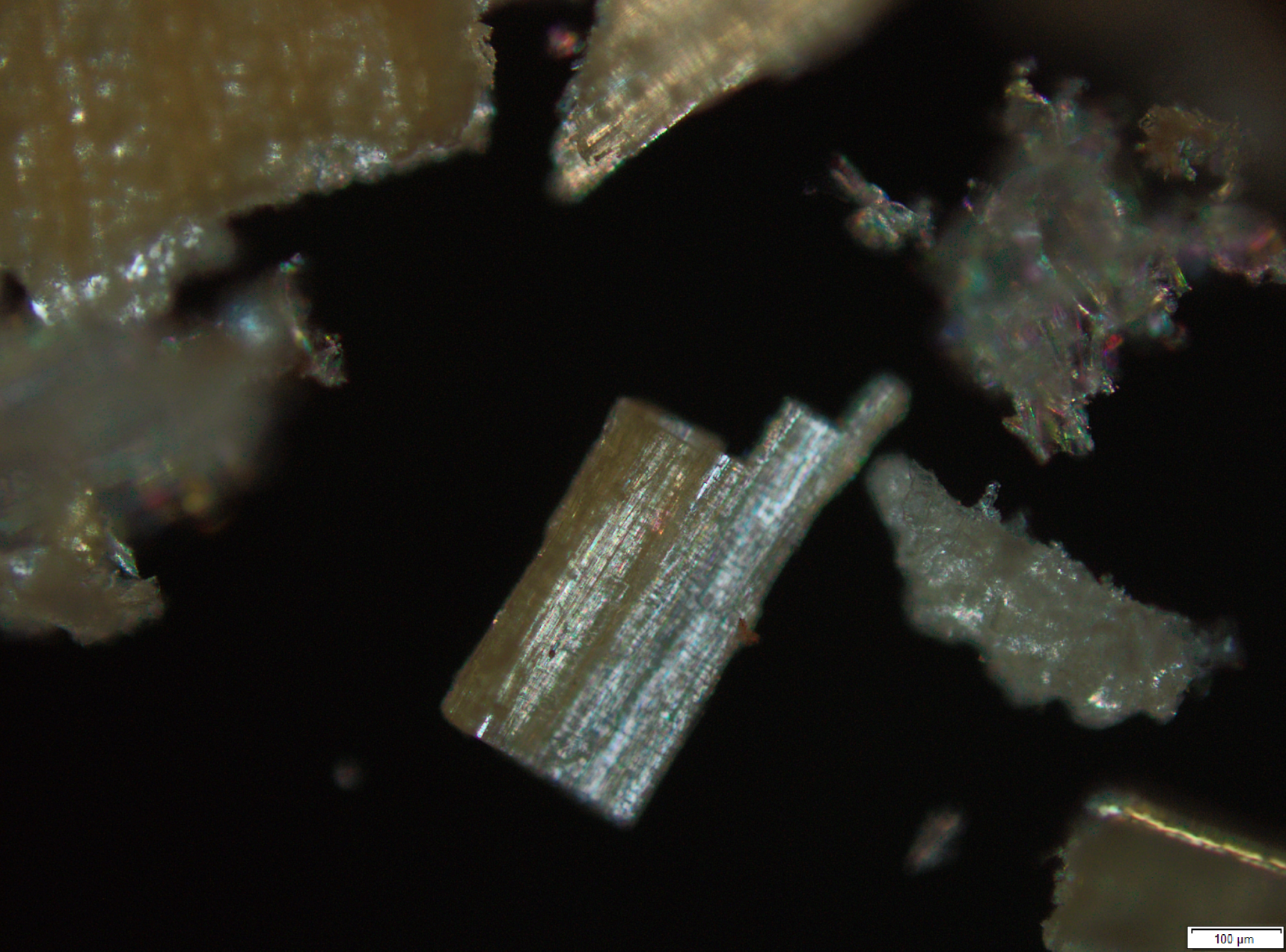

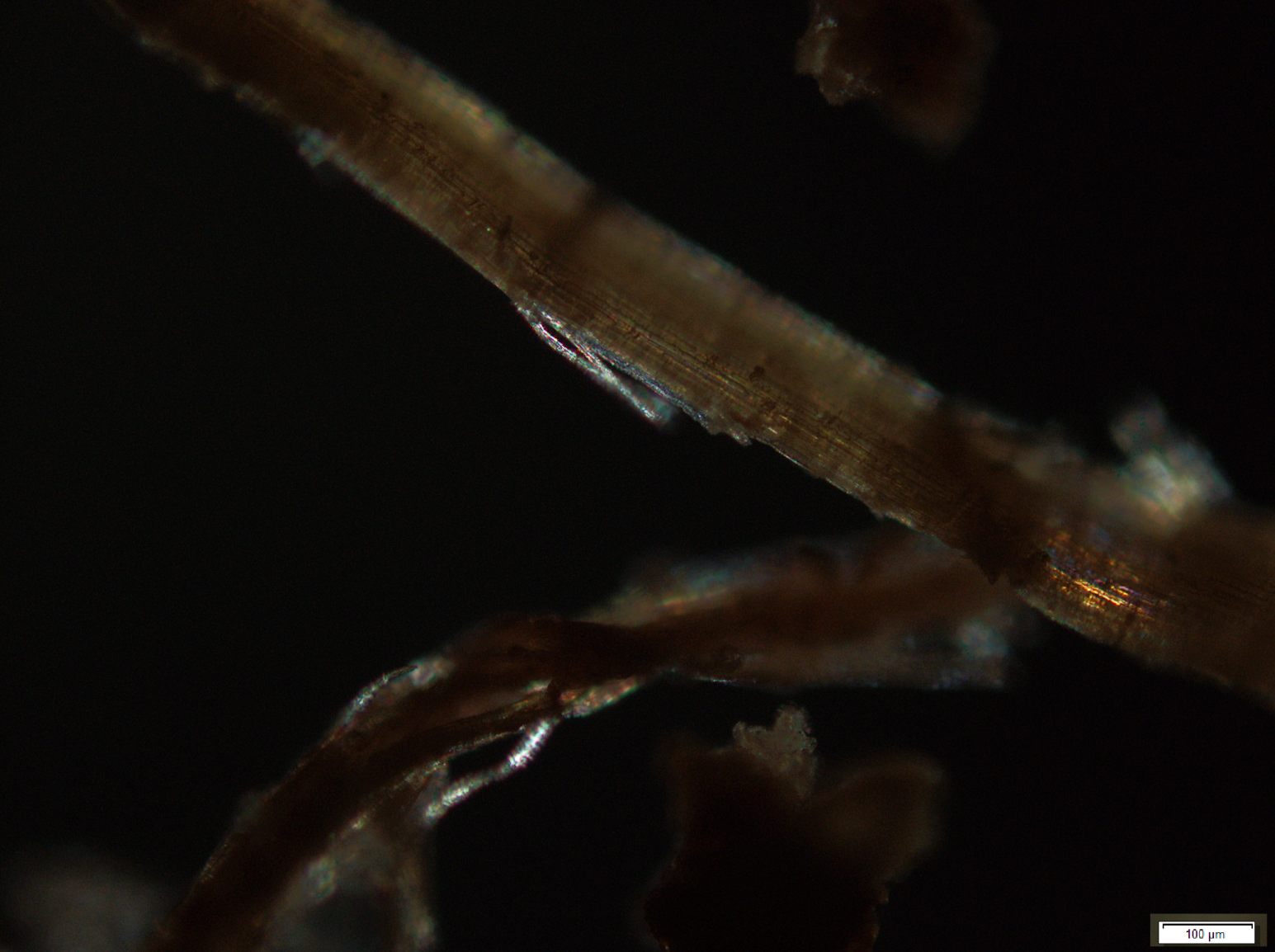

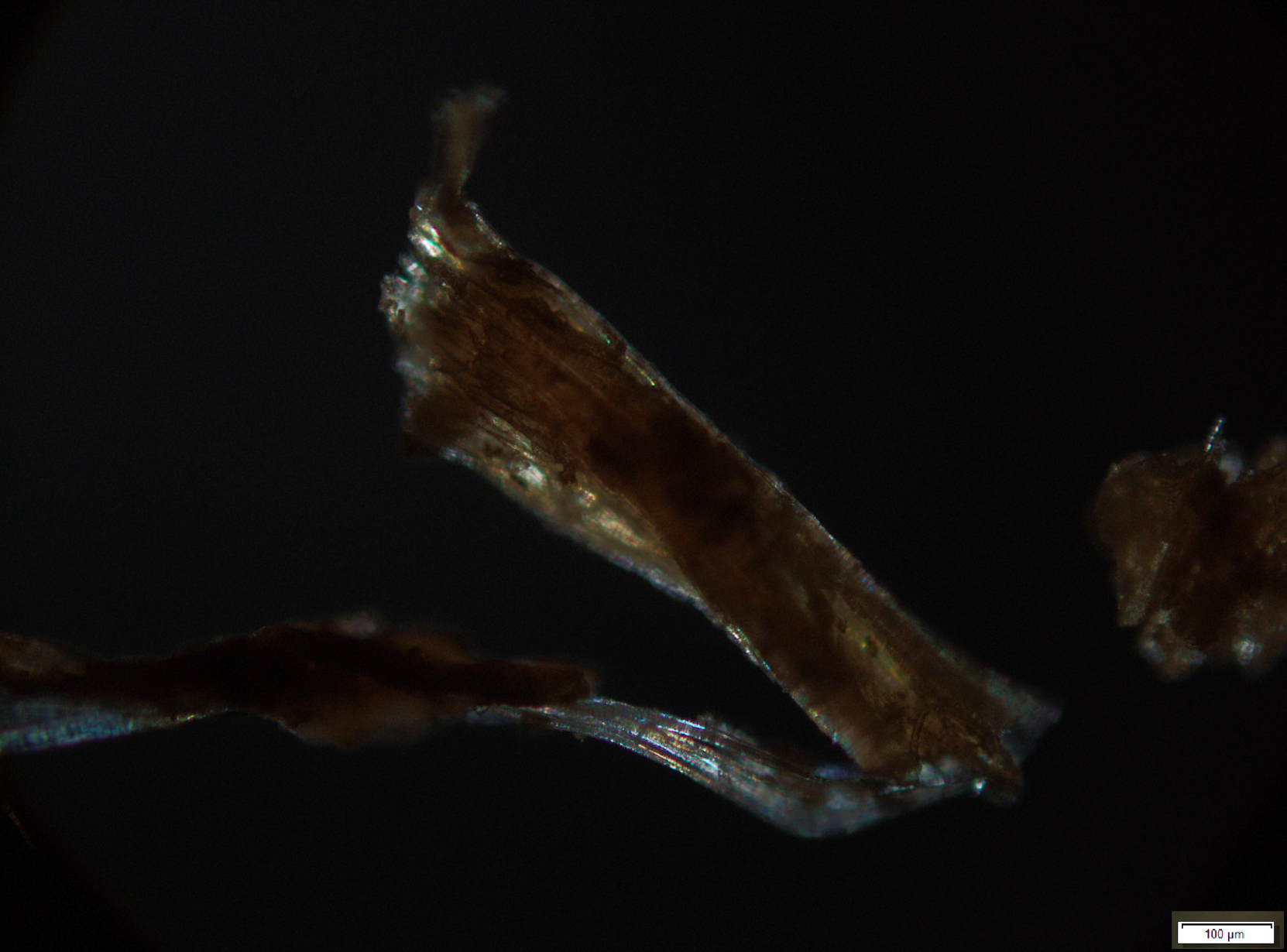


F**igure S2:**

**Figure S2:**  Low-resolution SEM images of (a) untreated; (b) DA-pretreated and (c) DA/Fe-pretreated samples and (d) higher resolution SEM image of lignin-rich globules extruding from a crack in a fibril of a DA/Fe-pretreated sample. Scale bars are 100 μ for (a), (b) and (c) and 1 μ for (d).


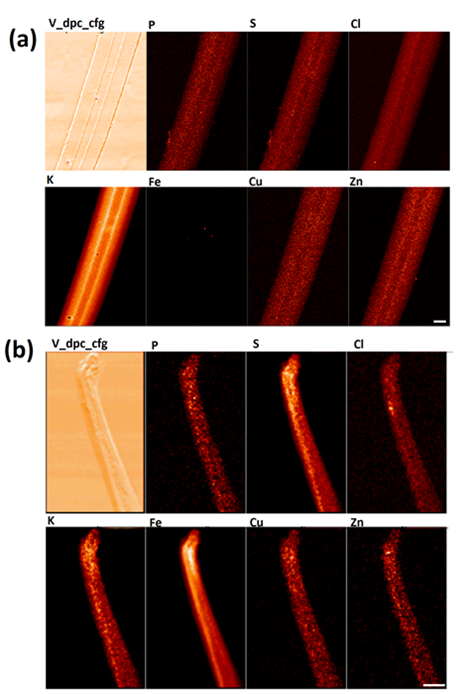


**Figure S3**

**Figure S3**  XFM images of fiber cells teased from (a) untreated and (b) DA/Fe-pretreated corn stover. V_dpc_cfg designates a differential phase contrast image. Distribution of seven elements are included. Iron is distributed uniformly throughout the cell wall of the DA/Fe-pretreated fiber cell supporting the USAXS observations that indicate extensive perfusion of iron throughout the lignocellulose. The iron and sulfur are largely exogenous due to the DA/Fe-pretreatment (it is very nearly absent in untreated or DA-pretreated samples). Other elements are endogenous and imaged at their natural abundances. Scale bars are 10 μ.

**Figure S4**


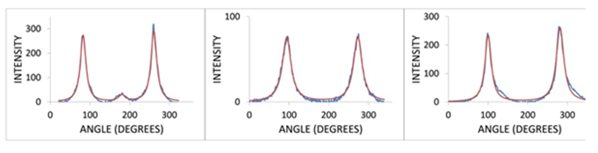


**Figure S4** Scattering intensity in USAXS patterns as a function of angle about the center of the diffraction patterns. Untreated (left) DA-pretreated (middle) and DA/Fe-pretreated (right) samples are shown. The strong peaks arise from the equatorial spikes of intensity that dominate the USAXS patterns. The weak peak observable at 90^o^ in the untreated sample comes from the characteristic diamond shape of background seen in untreated material but not in any of the pretreated samples. Pretreatment appears to result in little change in the distribution of orientations of the structural elements giving rise to these reflections.


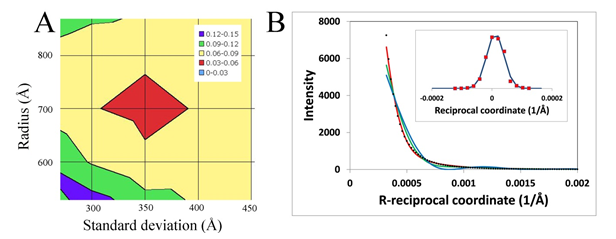


**Figure S5** (A) Two dimensional contour plot of residual (R-factor) between the observed and calculated intensities as a function of radius (vertical) and standard deviation (horizontal) of the solid cylinder model to account for the equatorial streak intensity in the DA/Fe-pretreated sample. (B) Calculated and observed (indicated as black dots) equatorial intensities for this sample. Both curves were normalized so that the area under the curve was one. The intensity (**red**) was calculated for the solid cylinder model of a radius of 700 Å with a standard deviation (SD) of 350 Å. The R-factor was 0.052. For comparison, two other calculated intensities (**green** and **blue**) were shown, i.e. solid cylinder (**green**) having an average solid cylinder radius (700 Å) and its SD (150 Å) and the single solid cylinder (**blue**) having a radius (700 Å) with no standard deviation. (B inset) The intensity distributions as a function of reciprocal coordinate (1/Å) for the direct beam in the equatorial direction. The calculated curves were derived by fitting a Gaussian curve having the integral width of 7.18 x 10^-5^ 1/Å to the observed intensity.


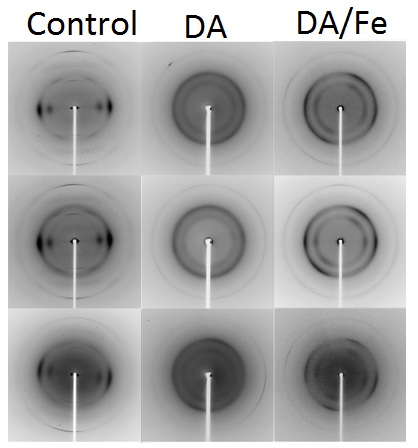


**Figure S6:** Representative wide angle x-ray diffraction patterns for control and pretreated maize.

*
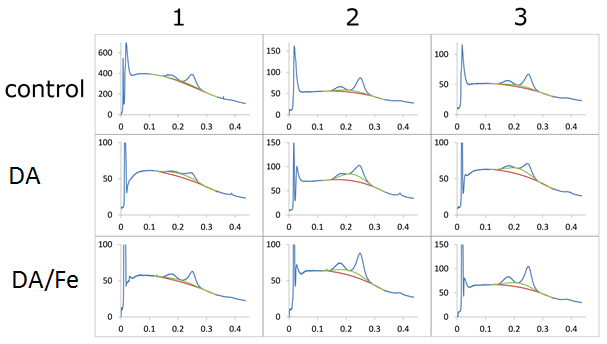
*

**Figure S7:** Equatorial intensity distributions as a function of reciprocal coordinate: The horizontal axis is 1/d = 2 sin θ/λ where $d$is Bragg spacing, $2\theta$is scattering angle, and $\lambda$is x-ray wavelength. The intensity was angularly averaged by FIT2D and plotted as a function of reciprocal coordinate in the lateral direction of cellulose chains. The fan shape region was selected after masking the region containing meridional and off-meridional cellulose reflections. One background tracing (**red**) shows a polynomial fit to the intensity data outside of the two principal equatorial peaks, while another tracing (**green**) shows a fit to the intensity minimum between the peaks as well as the intensities outside the peaks. The difference between these two background curves may correspond to the diffuse scattering arising from amorphous cellulose fibrils.


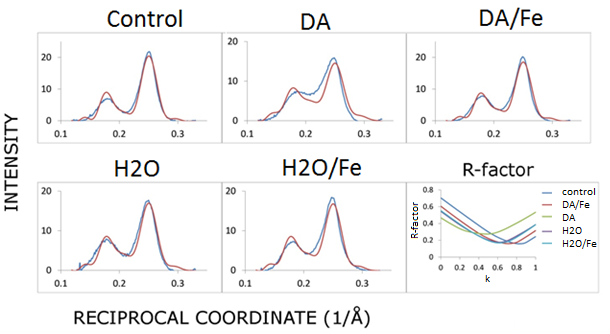


**Figure S8.** Observed (blue) and calculated (red) intensity distribution giving the minimum R-factor for control and pretreated maize samples. The two basis sets were 36-chain model as a crystalline domain, and 6-chain two layer model as an amorphous domain (see **Figure 3b**). The crystallinity, k, resulting in the lowest R-factor is tabulated in **Table S3**. The R-factor is shown as a function of k coefficient.
